# Positive Wild Animal Welfare

**DOI:** 10.1007/s10539-023-09901-5

**Published:** 2023-03-12

**Authors:** Heather Browning, Walter Veit

**Affiliations:** 1 University of Southampton, Southampton, England; 2 University of Bristol, Bristol, England; 3 Centre for Philosophy of Natural and Social Science, London School of Economics and Political Science, London, England

**Keywords:** wild animal welfare, positive welfare, sentience, measurement

## Abstract

With increasing attention given to wild animal welfare and ethics, it has become common to depict animals in the wild as existing in a state dominated by suffering. This assumption is now taken on board by many and frames much of the current discussion; but needs a more critical assessment, both theoretically and empirically. In this paper, we challenge the primary lines of evidence employed in support of wild animal suffering, to provide an alternative picture in which wild animals may often have lives that are far more positive than is commonly assumed. Nevertheless, while it is useful to have an alternative model to challenge unexamined assumptions, our real emphasis in this paper is the need for the development of effective methods for applying animal welfare science in the wild, including new means of data collection, the ability to determine the extent and scope of welfare challenges and opportunities, and their effects on welfare. Until such methods are developed, discussions of wild animal welfare cannot go beyond trading of intuitions, which as we show here can just as easily go in either direction.

## Introduction

The welfare of wild animals has been the subject of a recent surge of attention. Historically, although a few authors noted the occurrence of suffering in nature, particularly regarding predation, the prevailing view was that we should not intervene in the lives of wild animals, either because they were considered to fall outside our sphere of ethical concern, or because we could not expect such interventions to be successful (e.g. Regan 1983; Singer 1973). More recently, this view has been challenged, with authors arguing both that suffering is far more prevalent than the traditional view assumes and that we have duties to intervene where we can (e.g. Ng 1995; Horta 2015; Johannsen 2020b). There are thus two big questions that shape the current discussion on wild animal welfare: Firstly, what is the welfare status of wild animals? And secondly, how should we intervene to assist them?

The latter question has been engaged by both ethicists and scientists. Animal ethicists have worked to determine what our moral duties toward wild animals may be (Johannsen 2020b; Keulartz 2016; Kianpour and Paez 2021; Palmer 2013; Soryl et al. 2021), though as we will show, this work has often rested on problematic and speculative assumptions (see also Veit & Browning 2021a). Whereas the fact that some moral theory implied a duty to intervene on behalf of wild animals had previously been used as a reductio ad absurdum to suggest a flaw in the theory itself, others are now instead taking more seriously that this could be a legitimate conclusion (McMahan 2015). Due to a recognition of the welfare needs of wild animals and the growing dominion of humans over the ecosystem, the distinction between animals in the wild and in captivity is slowly disappearing when it comes to moral concern. Yet, while many now accept the moral call to protect the interests and welfare of animals, wherever they may be, some deny that the protection of wild animal welfare is feasible in practice, and thus not something that requires attention. This, of course, is an empirical claim still in need of scientific investigation, with current discussion largely resting on intuition - a point we will return to in Sect. 5. However, this subject is not the focus of our paper. Instead, we engage with the first question, of what the actual state of welfare is for animals living in the wild. This is the scientific question of wild animal welfare.

We need to know how good (or bad) the lives of wild animals are, and what conditions are having the greatest impact, before we can start to consider if or how we should intervene. To put it succinctly: we first have to answer what their welfare ‘is’ before we can make judgements about what it ‘ought to be’. In particular, we challenge the now-prevailing view that the lives of wild animals contain more suffering than pleasure; that the balance of wild animal welfare is net-negative (Horta 2010, 2015; Johannsen 2020b; Ng 1995; Tomasik 2015; Faria forthcoming). This view is now taken on board by many and frames most of the current discussion; but we think it needs a more critical assessment, both theoretically and empirically. In this paper we will examine this claim and the evidence offered in support of it to show that an account of net-positive wild animal welfare is at least plausible given the evidence, particularly with a more detailed consideration of the range of pleasures that may be experienced by wild animals. However, as we will emphasise throughout, this is not a question that can be settled by pure armchair philosophy, but rather one that requires more empirical data regarding the lives and experiences of wild animals. There is a need for measurement of the welfare of wild animals of different species and life stages, to draw accurate conclusions. Otherwise, all we have are our own, potentially unreliable, intuitions about the possible features of animal experiences.

Here, we follow the recent appeal for greater scientific attention to wild animal welfare, such as in the call for the establishment of the disciplines of welfare biology (Ng 1995; Soryl et al. 2021), and conservation welfare (Beausoleil et al. 2018; Beausoleil 2020; Learmonth 2020). Welfare biology is more directly intended as a naturalist approach to animal welfare that seeks to use the methods of a range of biological sciences, such as animal welfare science, behavioural ecology, and evolutionary biology, to assess the previously overlooked subject of the welfare of wild animals and extend our focus from animals in captivity to all animals. While there has been much scientific work on wild animals, this has primarily focused on their ecology, behaviour, and conservation, with scant attention paid to what their lives are like from their own point of view, i.e. their subjective experience.

Though there are many ways of understanding animal welfare (Veit and Browning 2021b); here we follow the dominant tradition in the wild animal welfare literature in taking animal welfare to consist in the subjective experiences of animals (see also Browning 2019, 2020). Animals outside of captivity are individuals, like all animals, and beyond simply being ecosystem actors, also have lives in which they seek out pleasure and try to avoid pain. It is important for the study of wild animals to look to find the best methods for discovering what this balance between positive and negative feeling, or ‘affects’, may be for different species, and how we might help in removing sources of suffering and promoting sources of pleasure.

The current literature is certainly right to challenge traditional assumptions that wild animals have high welfare resulting from their ‘flourishing’ in their evolved, species-typical manner (see also Browning 2019). The dominant public conception of wild animals still appears to be one of a more ‘idyllic’ state of nature (Faria and Paez 2015; Waldhorn 2019). It is common for people to take wild animals to have good lives, a viewpoint which forms the basis for a lot of the resistance to housing captive animals (Browning and Veit 2020, 2021). But these views are often based on intuition, rather than actual empirical evidence. As many have pointed out, and as we will see in this paper, there are many sources of suffering that wild animals face. Unfortunately, this has in many cases led to them going too far the other way, exaggerating the prevalence of suffering. While this may be important to encourage interest in the subject, and to counter the previously received view, it is also dangerous to work on misinformation if we want to make a real difference in animal lives. Where researchers such as Horta (2010) take their project to be one of debunking the ‘idyllic’ view of nature; here we take the opposite approach, to (at least partially) debunk the ‘hellish’ view that seems to have replaced it.

There are three primary lines of evidence that are used to illustrate the dominance of suffering in nature: the brutish nature of wild animal deaths, the prevalence of negative experiences within their lives, and the suffering taken to be attendant with reproductive strategies that involve producing large numbers of short-lived offspring (Horta 2010). In this article, we will tackle each of these in turn, providing an alternative picture to support the claim that most animals may actually have lives with net-positive welfare, or what would be considered ‘lives worth living’. Section 2, ‘Bad Deaths’, challenges the assumption that deaths in nature contain extreme amounts of suffering. Section 3, ‘Bad Lives’, challenges the assumption that wild animals necessarily have a low daily quality of life. Section 4, ‘Reproductive Strategies’ challenges the assumption that the dominant life-history strategy of r-selected species must necessarily involve a lot of suffering. After having discussed the evidence base for wild animal welfare, Sect. 5, ‘The Intervention Question’, will look at the issue of whether animal welfare interventions in the wild are feasible, arguing that consideration of the range of positive welfare experiences should lead to even more caution in intervention. Section 6, ‘Conclusion and Further Directions’ summarizes the key points of this paper, emphasising that more data is urgently needed to settle the matter and allow us to move forwards with planning effective strategies for assistance where required and suggesting further directions for the study of wild animal welfare; both for the purposes of ethics and our understanding of the lives of other sentient animals.

## Bad deaths

The first line of evidence that is often used in support of the predominance of suffering in the wild is the extreme pain and suffering surrounding animal deaths. In particular, predation is frequently taken as the paradigm case of the worst suffering that the wild has to offer – animals that are chased down by predators before being torn apart and eaten, often while still alive, creating states of fear and of excruciating pain (see e.g. descriptions in Tomasik 2015 and Soryl et al. 2021). McMahan (2015) presses the point, giving a vivid description of the “continuous massacre” and “unceasing mass suffering” caused by predators “stalking, chasing, capturing, killing, and devouring their prey” through “dismemberment, asphyxiation, disembowelment, poison, and so on” (McMahan 2015 p. 268). Such intuitions are widespread and evocative from wildlife documentaries, but that of course does not mean that this picture is necessarily true. In this section we argue that bad deaths will not count as much toward the overall balance of lifetime welfare as is often assumed, both because the duration is short and the intensity of suffering is significantly lower than it may first seem.

Firstly, the experience of suffering during death may not be as intense as one may think. Take predation – the prey experiences the pain of capture, killing and consumption. While anecdotes of animals suffering for days after an attack before their subsequent death are easily stored in our memory, most animals in the wild are killed quickly once they are caught, precisely because they might otherwise escape. They are often dispatched with a bite to the skull or nape of the neck and thus would experience minimal pain. It is true that some animals, such as African wild dogs or hyaenas, will catch and consume their prey while still alive, ripping out entrails and chewing on limbs as the animal dies more slowly from the injuries (Dawkins 1980 p. 52). This certainly sounds horrific and is not what any of us would envision as a humane death, but it may not be representative for most deaths taking place in the wild.

Furthermore, we should take seriously the possibility that these animals experience little pain at the time of death, due to a shock response. This experience has been reported in humans who have been severely injured in accidents. While they can recognise the extent of their injuries, it often takes time for the pain to begin. For example, Livingstone described his experience of being caught by a lion as ‘dreamy’, only aware of his injuries after his escape (Bostock 2003, p.85). This may explain the observed response in many animals captured and consumed by hyaenas: “it is rare that the victim puts up any significant active defence” (Kruuk 1972, cited in Dawkins 1980, p. 52). McMahan (2015) notes this, and that human reports vary widely – some will indeed report shock or unconsciousness that prevents pain, while others report extreme pain and terror. As he concludes, there is thus likely to be variation in the intensity of pain experience for prey. He adds that the evolutionary function of pain likely tips the scales toward pain experience in these cases, however we contend that the opposite is more likely.

The primary function of pain appears to be to motivate avoidance and recovery, triggering behaviours that may actually interfere with defensive and escape responses. If this is the case, then is only after the fact that pain would be functionally useful. More so, it would be fitness-reducing if the animal could not use its cognitive capacities to escape in a life-or-death situation. While it is certainly true that “evolution has no reason to prevent death from feeling unbearably awful” (Tomasik 2015, p. 136), if it is beneficial to allow animals to experience shock to facilitate escape, this may then also have the side-effect of benefitting dying animals. This is a form of ‘shock-induced analgesia’ that uses adrenaline and endorphins to temporarily block pain (Amit and Galina 1986). Some experimental evidence may support this: that activation of the fear system inhibits the pain response through production of endogenous analgesics. Rats exposed to predators (cats) show opioid-mediated analgesia, demonstrating reduced sensitivity to noxious stimuli, that is reversed with the opioid antagonist naltrexone (Lester and Fanselow 1985). This mechanism will then reduce the intensity of suffering for prey animals during death. We are not here trying to make a strong claim regarding the intensity of experience of animals during death – this is something that can only be established empirically. We are only trying to show that there are reasons to believe it may not be as severe as some would claim based on introspection and intuition alone.

Even when it is the case that the end-of-life states involve intense suffering, we should still not rank them too highly for their influence on lifetime welfare. The duration of such experiences is short, relative to the totality of an animal’s life experience and thus it is likely to be the case that the quality of the ‘average’ day is the biggest determinant of overall welfare (we will address the case of short-lived animals in Sect. 4). Unless the general life quality is very close to zero and the death is extremely painful and drawn out, it is unlikely to outweigh overall. Take predation again: where deaths by predators are described as being slow, they are still in the scale of minutes rather than hours. McMahan (2015) describes them as “a quarter of an hour or more” (McMahan 2015, p. 279), which is undeniably an unpleasant length of time, but not one that is likely to outweigh a length of life. The claim that “even if animals enjoy net happiness during most of their lives, this may be outweighed by the painful intensity of their deaths” (Tomasik 2015, p. 139) must therefore appear to be quite overblown. Emphasising short bursts of extreme pain can bias us to think of a life as worse than it is overall, influencing us to consider them as representative for a whole life experience.

Other types of deaths will be more prolonged and thus possibly a cause of more suffering overall – a lower intensity, but a longer duration. Typically, the longer the duration of the death stage, the lower intensity we would expect it to be – it is rare to find an excruciating prolonged experience. Deaths from starvation or illness may occur over days or even weeks, during which the animals will feel highly unwell, but even these are still short in terms of most animal lifetimes. This may be truer for larger animals with long lives than for small ones with shorter lives (as there are more opportunities for counterweighing positive experiences). These points should at least take much of the initial force out of the intuitively plausible arguments that death is major influence on lifetime welfare in most cases. Nevertheless, as we will emphasise throughout this article, actual research establishing the duration and intensity of suffering during death is needed to answer this question properly. Let us thus now turn to the question of whether the total life experience of an animal is itself bad.

## Bad lives

It can be claimed that even if we remove considerations of the pain and suffering at the time of death, there are still many sources of suffering during life that may lead us to think that these lives are not net-positive, or at least not to a degree that outweighs a painful death. Horta (2015) emphasises a range of ‘disvalues’ suffered by animals, including predation, parasitism, malnutrition, disease, injury, and unfavourable weather. Likewise, Tomasik (2015), Johannsen (2020b) and Soryl et al. (2021) describe a range of negative experiences faced by wild animals. We agree that there are many negative experiences that animals face every day: fear, disease, pain, thirst, and starvation, even smaller negative affects such as itches, among many others. However, what is not considered in these discussions are the range of positive experiences also available to wild animals, of which we take there to be many more than are commonly considered.

It is not enough to merely establish that there are many sources of suffering – we must also show that they are of a sufficient frequency and intensity to outweigh the positive experiences, and this requires describing and assessing both (a point also raised by Mikkelson 2018). However, the dominant literature, such as the papers listed above, seem to neglect this positive side of the equation. While the possibility of positive experiences is acknowledged for some animals, it is not investigated in detail and is presumed to be absent for many (the cases we will discuss in Sect. 4). We cannot here establish the balance one way or the other, without much more data regarding the daily conditions of wild animals and their impact on welfare experience. However, we give some reasons to consider that the balance may be more often positive than negative. Our strategy here is threefold – to show that some of the negative experiences may be less prevalent than claimed, to show that most will be offset by countervailing positive affects, and finally to suggest that there may be a positive baseline experience raising the net welfare balance.

Firstly, many negative experiences may not be as frequent or intense as some of the literature in this area would imply. A good example of this is fear. Fear is taken to be a huge source of suffering for prey animals (Soryl et al. 2021; Tomasik 2015). While it is almost certainly true that animals being pursued by predators experience an extreme fear (motivating the flight response), this will typically only last for a minute or two (Bostock 2003). However, it has also been claimed that prey animals live in a constant state of fear, watching and waiting for the next predator attack. The behaviour of prey animals is strongly determined by the presence of predators, effects known as ‘fear ecology’ (Ogden 2016; Zanette and Clinchy 2019) and the ‘landscape of fear’ (Laundre et al. 2010). Prey animals will reduce breeding efforts and change foraging patterns and habitat use in environments with predators (Laundre et al. 2010; Ogden 2016); effects which will disappear when predators are excluded, indicating they must have a proximate mediation (Ogden 2016). Animals will suffer behavioural, physiological and neurobiological costs that may decrease their welfare (Zanette and Clinchy 2019). Studies on the effects of prolonged predator exposure on laboratory rats and mice have demonstrated that this can result in strong stress and anxiety responses (both behavioural and physiological), that shows slow habituation (Belzung et al. 2001; Blanchard et al. 1998) (however, it is worth keeping in mind that these last are studies of captive animals with very little control over their escape options - Belzung et al. 2001 even emphasise that it is exposure to “unavoidable” predators they have tested - and thus we must be cautious about the ecological validity).

There is no doubt that predator presence has strong effects on prey, but what is currently missing is the links to affect. It is not clear how many of these behavioural and physiological responses are associated with negative subjective experience. Behavioural changes could also be the result of caution – a risk-benefit trade-off calculation. While prey animals may suffer decreased welfare in the presence of predators, in terms of reduced opportunities, it does not necessarily follow that there is a constant feeling of fear. In fact, it seems unlikely that many animals live in a state of chronic stress; it is certainly not adaptive. Stress responses are bad for organisms – they interfere with other body processes, suppress the immune system and potentially even alter the epigenome of offspring – and for these reasons animals are likely to minimise them (Ginsburg and Jablonka 2019; Sapolsky 2004). We need measures of subjective fear experience in order to determine whether or not this is actually a constant welfare challenge.

Although this is just one example, it serves to illustrate a more general point: we must be careful not to conflate the presence of stressors or harms with the experience of negative affects – evidence of a stress or disease response for instance is not sufficient to assume negative experience without additional evidence linking this to welfare, such as behaviour. While of course most stressors and harms will have some negatively experienced component, the degree and intensity of this cannot just be taken for granted.

Our second argument is that most non-lethal sources of negative experience will be balanced by counteracting positive experiences, and that the full range of these positive experiences is rarely considered, thus biasing the calculus in favour of negative welfare. The presence of balancing positive affects makes sense if we consider the functional role of affects. Most accounts of the evolution of valenced experience – the ability to experience positive and negative mental states – take affects to play a motivational and decision-making role in guiding animal behaviour (e.g. Cabanac 1992; Dawkins 1998; Spruijt et al. 2001, Veit 2022, forthcoming). Negative affects will motivate animals to move away from particular stimuli or seek methods of reducing the experience, while positive affects will reward animals for behaviours that are in their interests (Fraser and Duncan 1998). Frequently, these will be paired. For example, an animal deprived of food will feel hunger, a negative affect that motivates it to seek out something to eat, which is important for building and maintaining their body. However, there are also positive affects that reward the experience of eating – satiation (the feeling of fullness after a meal), gustatory pleasure (the enjoyable taste of preferred food) and even behavioural pleasures in seeking and processing the food, such as the satisfaction of hunting behaviours. There are a range of different types of comfort and satisfaction associated with meeting basic needs, that often go uncounted, such as thermal and resting comfort.

So while animals experience negative affects when things are going wrong, they will also have positive experiences when they correct for these, and these positives will cancel out at least some of the overall negative experience. Being cold feels bad, but curling up in a cosy den feels good. We need more information to determine the intensity and duration of all these positive and negative experiences to determine if, and by how much, one set may outweigh the other. It is true that evolution does not ‘care’ about welfare and there is no evolutionary reason for welfare to be maximised beyond motivating fitness-enhancing behaviour, such that there will be cases where the two come apart and fitness ‘wins’ (Soryl et al. 2021). However, we would expect that most often the two will work in tandem, else the potentially costly affects would not be preserved.

Finally, there are a large range of additional positive experiences that animals can have over their lifetime. Social bonds, affiliative behaviour, courtship, mating, and rearing offspring are all potential sources of positive experience. Young animals play a lot - and sometimes older animals join in - behaviours which are typically taken to be indicative of positive welfare (Held and Špinka 2011). Performance of many natural behaviours will be positively valenced - for example a recent study suggests that presence of endogenous opioids signals that birds find singing intrinsically pleasurable (Stevenson et al. 2020). So even beyond simply the satisfaction of bodily needs that weigh against the negative experiences of dissatisfaction, there are additional positives an animal can experience, associated with a variety of natural behaviours, that help shift the balance back in a positive direction.

In particular, we may consider that the ‘baseline’ experience of an animal is mildly positive. If we strip away all the specific physical demands, the experience of just existing could be a positive one – a “joy of living” (Ginsburg and Jablonka 2019, p. 189). This makes sense from an evolutionary standpoint – that an animal for whom existence is positively valenced is one more likely to value its own life and to seek its continuation (Humphrey 2011). It could even have formed the earliest types of experiencing, an “inner feeling of living which if experienced as positive, would drive behaviors that sustain it and lead to the evolution of what we call more specific ‘wanting’” (Ginsburg and Jablonka 2019, p. 189). This is not to imply that a sophisticated cognitive representation is needed, but merely that a positive feeling is one which an individual may fight to maintain.

This is also likely to be true of the basic acts of exploration and engagement with their environment – the ‘SEEKING’ system is one of the proposed core affects such that moving about and investigating new spaces and objects will be a source of pleasure (Panksepp 2005). Exploration provides the benefits of new knowledge and learning, and it thus makes sense for it to be motivated by associated positive affect (Ginsburg and Jablonka 2019). Associated with this are a potential range of perceptual and sensory pleasures, such as preferred visual, auditory, and olfactory stimuli. Again, if gaining information from the environment provides a fitness benefit, there is a plausible story to tell regarding the benefit of positive affects in motivating animals to seek out a variety of perceptual inputs. If this is true, then this gives us even more reason to think that the negatives don’t have to outweigh the positives.

One counter to this type of reasoning is to deny that we can compare positive and negative experiences in this way – either within a single animal or across different individuals. For example, Belshaw (2016) argues that positive experiences cannot compensate for negative experiences within the lifetime of an animal. This is not a claim that suffering is so bad that no amount of pleasure can outweigh it, but that for an animal, the lack of psychological continuity or integration over time means that moments of intense suffering make life not worth living for that animal, regardless of past or future pleasures. In reply, Višak (2017) shows that, on any plausible reading of this claim, it is not true. Pleasurable experiences should be able to compensate suffering for animals, just as they can for us – the value is not only a feature of the way our lives cohere. Harnad (2016) argues that we cannot aggregate experiences across different individuals – that “my orgasms cannot be traded off against others’ agony”. This is a stronger claim, that we cannot aggregate welfare across individuals at all. Of course, this is a claim against the very premise of a utilitarian calculus that underlies much work in animal welfare science and (human) happiness studies alike, and the defence of an entire ethical framework is not one we can attempt here. Within the debate we are engaging with, it is taken to be the case that such comparisons are allowable; though there may be additional difficulties in practice when attempting to compare welfare across individuals or species (Browning 2023). As is often the case, difficulty of comparison and measurement does not imply impossibility.

Again, we are not making any strong claims about the distribution of pleasures and suffering for wild animals. The intensity and duration of different experiences is likely to vary widely across species and can only be determined by careful investigation into the lives of different animals. What is crucial is that we examine not only the sources of suffering, but also the sources of positive experience, and how they are weighted by the animals. We have provided here some reasons for optimism that in many cases, this balance will be positive, in particular because there are a wide range of pleasures that animals can experience that are often not considered when thinking about their welfare states. Counting the more basic but widespread comforts and sensory pleasures, as well as the potential baseline positive affect, is more likely to tip the balance in favour of positive experience.

## Reproductive strategies

For those who argue that suffering prevails in nature, they may accept all that we have presented above and still hold that most animals will have net-negative lives. This is because the majority of animals born are of so-called ‘r-selected’ species, a fact that forms the basis of the primary argument presented by those in favour of the view we are opposing in this paper (e.g. Horta 2010, 2015). Johannsen (2020b) explicitly lays out the central argument (p. 12):

Premise 1: A life that’s filled with suffering and ends shortly after birth is not a flourishing one, and it may not be worth living.

Premise 2: Most r-strategists live lives that are filled with suffering and end shortly after birth.

Premise 3: Most sentient individuals born into the world are r-strategists.

Conclusion: Most sentient individuals born into the world do not live flourishing lives, and their lives may not be worth living.

In this section we will challenge this argument on two grounds. Firstly, we deny that this strategy does necessarily result in a large amount of suffering (denying premise 2), and secondly, by showing that even where it is the case, it is insufficient to outweigh the positive welfare in the lives of other surviving animals.

R-selection is a life-history strategy in which animals produce a very large number of offspring, with low parental investment, where most will die quickly but a few will make it through to the next generation. This is particularly common in smaller species, such as invertebrates, as well as fish, amphibians and reptiles. In contrast there are K-selected species, like humans, that produce a small number of offspring with high parental investment in each, to ensure a high rate of survival. While these strategies are part of a larger spectrum, most animals can be described as tending toward one end or the other. These strategies ensure that most animals born will be more toward the r-selected end, as a single parent can produce hundreds or in some cases even millions of offspring, with the expectation that only a few will survive. While a large proportion of these may not even survive to birth/hatching, it still leaves a large number that will be born and will die shortly after. This is taken to mean that most animals that ever exist will have short lives with a lot of suffering – dying quickly from predation, or starvation when competing for resources against their siblings and conspecifics. Their lives are taken to be almost entirely suffering, and thus the number of animals with net-negative lives will outweigh those surviving members who have positive lives (Horta 2010).

Related to this is the economic model created by Ng (1995), purporting to demonstrate that suffering must dominate in nature, based on evolutionary and ecological factors, particularly the fact of excess production of offspring. He takes it as given that animals that do not survive through to mating will most likely have negative welfare, though rather than providing any particular evidence for this claim, simply reflects that “it is difficult to imagine a positive welfare for such a life” (Ng 1995 p. 271). As we will discuss, even if this is true for the individuals, this does not necessarily mean that their negative experiences outweigh overall the positive experiences of their surviving relatives. Yet, many animal ethicists cited it as scientific ‘proof’ that animals in the wild will suffer and that we would require radical interventions. After all, Ng himself claimed that “if we can reduce the number of such miserable individuals, other things being equal; we can increase the level of over-all welfare” (p. 271). But as our previous sections demonstrated, such intuitions may well not be well-motivated.

Importantly, Ng’s model relies on the ‘Buddhist premise’, that proposes that under a set of assumptions about the costs of enjoyment and suffering, there will be an excess of suffering over enjoyment. This he takes to imply that the pleasures associated with successful action will be less than the suffering associated with unsuccessful action. This premise was revised in a later paper (Groff and Ng 2019), showing the original model to contain mathematical errors undermining his Buddhist premise. The authors admitted that the balance between positive and negative experiences may instead go either way, depending on the living conditions of the animal. The original model "when fixed, negates the original conclusion. Instead, the model offers only ambiguity as to whether suffering or enjoyment predominates in nature” (Groff and Ng 2019, p. 39). In particular, we need to better understand the production function of pleasure and suffering. Without further understanding of the costs and benefits of affective experiences within the contexts of each different organism, it is not possible to theoretically hypothesise which is likely to dominate. However, despite this reversal, much of the damage has been done, with the original model still being cited in favour of the claim of the predominance of wild animal suffering, with the revised model still being largely ignored.

The primary reason to doubt that this reproductive strategy creates overwhelming suffering is that the lives of those that die early may not be entirely filled with suffering. Horta (2010) claims that these animals will have “little (or no) happiness in their lives” (p. 81), but as we have discussed in the previous section, there are a wide range of potential positive experiences available to wild animals. In the short period of their lives, they may therefore also experience small pleasures of exploring, finding food etc., and not only dizzying fear, pain and hunger. Pleasure and suffering are not directly correlated with survival and reproduction, but with the different experiences associated with these (e.g. finding food, avoiding predation, avoiding disease and injury, finding social companions). It therefore does not follow that an individual who does not survive or reproduce will not have opportunities to experience pleasure – for instance, a young animal may have many instances of satiety, comfort, social bonding etc. Additionally, though Horta (2010) takes it to be the case that death by starvation or predation will be painful enough to outweigh any sources of pleasure, we have given reasons in Sect. 2 to doubt this. Individuals at very early life-stages taken by predators are likely to die quickly, without prolonged suffering. For animals with very short lives, this may not hold, but then neither will they have lives with very many experiences so even if all experiences are characterised by suffering, they will not amount to much in the overall total.

It is also possible that juvenile r-selected animals have reduced sentience (i.e. that their conscious experience is dim, and the high of their highs and low of their lows correspondingly reduced). It is unclear exactly at which stage of development sentience emerges, and this is likely to be different for different species – ranging from pre-hatching to several months after birth (Mellor 2019). Where larval forms have underdeveloped neural anatomy and sense organs, it is less likely that they will have the full capacity of an adult member of their species. Without a better understanding of the neurophysiology of sentience, this possibility remains open.

There is also the possibility that there is not an adaptive advantage for younger animals to develop full sentience. A complex brain is costly, and is only worthwhile if it provides sufficient benefit. If it is the case that large numbers of offspring will die early, and in cases where this will be through no fault of their own (i.e. there is mostly an amount of randomness as to which animals will perish and which will survive, rather than being a result of the decisions made and actions taken by the animals themselves) then there is less advantage for animals to develop the capacity to suffer at this stage. As pointed out by Groff & Ng (2019), there is the possibility that as the number of doomed offspring increases, the suffering of each decreases, as the costs of producing suffering would be unlikely to benefit the organisms in these circumstances. Again, this is speculative and would require further research into the adaptive function of sentience, however is a potential way in which the net suffering of juvenile r-selected animals may be lower than often claimed. Until this is better understood, it cannot be taken as strong grounds for supporting the argument for net suffering in wild animals. While Horta (2015) considers this possibility, he takes it that the overwhelming numbers of such individuals would still lead to net suffering. However, we think that with the additional considerations of less painful deaths and more positive life experiences than is often allowed, there is less reason to be confident in this conclusion.

We have given reasons to suggest that both the intensity and duration of suffering could be lower than assumed for r-selected species, and this may therefore mean that their suffering will not dominate calculations of wild animal welfare. But even if we grant that these animals do suffer a lot, this does not necessarily support the conclusion that suffering dominates. The argument given is that pure numbers cause the suffering to swamp the pleasure, even if we took the assumption that those who survive do go on to have good lives (which, as addressed in the previous sections, is not often granted). However, the problem with this argument is that it seems to conflate number of lives with amount of suffering. It is not the case that ‘the majority of wild animals experience net suffering’ is equivalent to ‘suffering prevails in the wild’. Even if one were to grant that there were this overwhelmingly large number of individuals with negative lives, it does not have to entail that there is overall more suffering than pleasure, and this is because the length of lives differ so significantly.

Take the example used by Oscar Horta (2010), an Atlantic cod. These cod can produce up to two million eggs, but if the population remains stable then on average only two will survive to adulthood. He further stipulates a 10% chance of the eggs surviving to hatch, a 10% sentience of the offspring (he states it as a 10% chance of sentience, but we may also see it as having 10% of the sentience of the adult) and a life that nets 10 s of suffering before death (with perhaps the rest of life being neutral). This will still create 200,000 s of suffering, which is the equivalent of around 2.3 days. Thus we would need to offset 2 days of intense suffering to avoid a net-negative outcome. The two surviving individuals will have on average a lifespan of 16 years, or 5840 days. If these individuals live a life that is only just barely positive (say overall 0.1 out of 100), then even if we take the suffering to be extreme (100/100) then we still end up with the pleasure outweighing the suffering by over 8x, simply by virtue of length of life. Of course there may be cases where these numbers can be set such that the suffering will outweigh the pleasure, but it is at least not immediately obvious and many cases are instead likely to turn out the way we describe.

Many r-selected species will thus have sufficient positive welfare for their surviving members to outweigh the suffering of those that die. Additionally, the K-selected species will not be subject to this effect and so most individuals will not have lives dominated by suffering (dependent on how we take the considerations from the previous section). We need to make more direct comparisons based on the number of episodes of pleasure or suffering that the different individuals have, and whether the much longer lives of the surviving individuals (and the K-selected species) will outweigh the shorter lives of the unsuccessful r-selected individuals. In this section we have provided reasons to think that they often will, and thus that pleasure rather than suffering will dominate in nature.

## The intervention question

We have provided several reasons to think that wild animal welfare is far less likely to be net-negative than the common view would suggest. The question that remains is what difference this would then make in discussions about possible interventions to improve wild animal welfare. We think there are two types of cases worth distinguishing, and which will be affected in different ways: decisions about interventions affecting which and how many animals will exist, and interventions aimed at reducing suffering for those animals that do exist.

The primary upshot to the difference between the claim that wild animals have lives that are overall net-negative versus that they are net-positive are changes in how we calculate the costs and benefits of actions that result in the loss of many wild animal lives, i.e. when we engage in population ethics for non-human animals. For example, if nature is dominated by suffering, we may think that we should take drastic actions such as reducing the numbers of wild animals (Belshaw 2016) and even reconsider actions that may typically be morally criticised, such as land clearing, to be instead a welfare-enhancing action (Tomasik 2017). If suffering is what we want to eliminate in a world where suffering outweighs different kinds of pleasures, our policy may be to prevent the birth of wild animals, rather than to improve their lives. Indeed, we might even think that conservation efforts are detrimental to animal welfare, insofar as they increase the number of suffering animals. The idea that the net-negative welfare of wild animals could entail that their removal would be an improvement has been called the ‘logic of the logger’ (John and Sebo 2020). As well as changing the expected value of actions like these, it will also influence how strong a priority we place on wild animal welfare in comparison to other areas of animal welfare concern – such as whether this is a more urgent matter than factory farming.

This is not to say that an acceptance of net-negative wild animal welfare must necessarily lead to these conclusions – we may think, for example, that destruction of habitat would be undesirable because of environmental values or that our negative duties not to harm outweigh our positive duties to intervene (Johannsen 2020a). It is not unreasonable to think that such policies would make our actual treatment of animals worse, for instance, by having negative effects on human attitudes toward animals (John and Sebo 2020) or if we disrupt habitats such that the species balances change, favouring r-selected over K-selected species (Johannsen 2020a).

As noted by Višak (2017), recognition of suffering can be addressed either by reducing the sources of suffering or the bearers of suffering. Where one thinks that animals have lives of net suffering, they may be drawn toward the latter option, particularly if they think it would be difficult to address the former. This might then lead to ranking the best options as being to kill suffering animals (as we would in the cases of humane euthanasia), and/or to prevent them coming into existence in the first place. In this paper we have shown that there are reasons to doubt that wild animal lives are predominately suffering, and thus the latter would not be an ideal strategy. However, importantly, we take it to be the case that even if wild animals did have net-negative lives it would not follow that these would be the best actions. It is still better where possible to reduce or prevent suffering than to remove its bearers – if we succeed in the former we would have created more value, rather than simply removing disvalue. Thus, interventions aimed at reducing suffering or improving wild animal lives will have a greater overall expected value than those aimed only at reducing total numbers of wild animals.

This then leads us to the second case - interventions aimed at reducing suffering. After all, if we all agree that there is a lot of suffering in the wild, does this not still provide reasons to intervene, regardless of which way the overall balance sits? Regardless of what we think about the net balance of welfare for any animal species, it is undoubtedly true that there are many sources of suffering and welfare could potentially be increased. If we identify those processes creating the highest amount of suffering, we can determine where we may be able to intervene to improve wild animal lives. For this, we need to better understand welfare, how affect operates in natural settings, and the range of experiences different individuals undergo in their lifetimes. Wild animal ethicists are also right to point out that we need to bring wild animals into our deliberations, even if we end up deciding not to intervene. Whether or not it dominates, there is a large amount of suffering in nature, and this is not something we should just ignore.

This leaves the issue of feasibility, perhaps the greatest challenge to wild animal welfare, and one we argue is even more complex once a full consideration is included of the range of pleasures available to wild animals. Even if we identify sources of suffering, and decide that this should be of moral concern to us, we may simply lack the ability to intervene in a controlled or predictable way due to complex interactions and unintended effects. We may be unable to even predict whether our actions will lead to a net increase or decrease in wild animal welfare (Delon and Purves 2018). When we consider the wide range of potential positive experiences that could be altered or removed, our confidence in having an effect that is beneficial overall should be decreased.

Indeed, our history of ecological interventions has been for the most part a worrying one. Ecological systems are highly complex and interconnected, and small changes in one area can lead to large unintended effects elsewhere. However, we take this as a reason for caution, not for inaction, and definitely not a reason to dismiss ethical concern for wild animals. Instead, it can be seen as another reason to further ecological research, alongside conservation and welfare biology. The more we understand about our ecosystems, the better our chances of making welfare improvements. In the end, again, it comes back to a need for better understanding of wild animals. In the meantime, we could adopt something like ‘fallibility-constrained interventionism’ (Johannsen 2017) or the ‘interventionist thesis’ (Torres 2015); advocating that we should aim to intervene but only where we have sufficient understanding of the relevant ecosystems and will not create more suffering. Only if we think the problem is entirely intractable – that there is no way we could ever obtain enough information to reliably predict the outcomes of our interventions, particularly the range of indirect effects (or at least with a permissible degree of uncertainty) – would we think we should never intervene (Delon and Purves 2018). This will be more likely for large scale than for small scale interventions, but we think this level of pessimism is unwarranted overall. We thus advocate a cautious, rather than eliminativist, strategy for intervention.

There are a range of potential proposed interventions for wild animal welfare, carrying different levels of risk. Some of these operate at a smaller scale and are already in place, such as vaccinations against disease outbreaks, rescuing animals from natural disasters, rearing orphans and supplementing food during times of shortage (Faria and Paez 2015; Horta 2017). We take these as least likely to pose an overall welfare risk. However, there are other more extreme proposals that need to be approached with greater caution, such as gene editing to reduce the breeding rate of r-selected species (Johannsen 2017), altering or removing predators to prevent predation (Bramble 2020; McMahan 2015; Pearce 2015b), eliminating parasite species (Johannsen 2020b), genetically modifying animals to adapt to the consequences of climate change (Palmer 2016), high-tech ‘stewardship’ of entire species (Pearce 2015b), even through to a wholescale modification or redesign of natural systems – what has been termed ‘paradise engineering’ (Kianpour and Paez 2021) or utilising genetic engineering and nanotechnology as pathways to alter the neurological processes of all sentient life with the aim of abolishing all suffering, and enhancing positive experiences (Pearce 2015a). Such complex interventions are far more likely to hold unexpected side-effects, including removal of positive welfare experiences, and need to be thoroughly investigated before implementing.

The feasibility and desirability of different interventions will therefore depend on the specific context, and understanding of the processes involved - for example, restoration of habitat or reducing pesticide use being probably far more feasible than preventing predation. Measures such as promoting concern for wild animals and changing the default assumptions regarding our lack of duties toward them, are some other methods that can encourage change without necessarily causing great harm (Horta 2017; Tomasik 2015). However, it is important to keep in mind that any changes may have unintended consequences, and a failure to adequately recognise the range of positive experiences in the lives of wild animals means these consequences are more likely to be harmful, potentially depriving animals of previous sources of positive welfare. This is not to say that no such interventions should ever be attempted, but merely to add an additional layer of caution; that the problem of negative unintended consequences may be even greater than it first appears once positive welfare experiences are included in the calculation.

## Conclusion

In this paper, we have shown that the assumptions underlying the claim that suffering dominates in nature are not always justified, and thus that it is at least as plausible that pleasure dominates. However, as we have emphasised, this is a matter that requires empirical data to determine the extent of wild animal suffering. Like Soryl et al. we consider it an “open question whether the life of a wild animal is worth living” (2021, p. 11); though we are more optimistic about the answer than they appear to be. We do not doubt that other researchers in this area would all agree that more research is needed to properly answer questions about wild animal welfare. Where we differ is in our prior expectations of the likelihood of a finding of a net-positive or net-negative welfare balance for wild animals. For instance, Horta (2010) takes it to be the case that we can make “informed guesses” about the lives of wild animals, that can be “well-grounded in our knowledge of relevant facts about what happens to them in their lives” (p. 76). We agree, but as we hope to have shown in this paper, it is important to keep in mind all relevant facts, particularly including investigating and counting positive welfare states alongside the negative ones so commonly discussed. This mirrors a shift in focus within animal welfare science more generally - where it was originally almost exclusively concerned with reductions of suffering, there is now also a focus on creating positive experiences for other animals (Yeates and Main 2008).

There are biological reasons weighing both for and against a model of predominant suffering in nature, and without further data, we end up merely trading intuitions. While no-one denies that there are many sources of suffering for wild animals, there are also many sources of pleasure, and we cannot from the outside try to weigh these against one another. There are many reasons why our intuitions may be faulty – we may fail to empathise with the exact experiences of wild animals or we may only think about small sub-sets of existing animals (with a bias toward the more visible larger vertebrate animals) (Horta 2010; Tomasik 2015).

Which types of experience an animal has, their intensities, and their durations, will all be important sources of information to consider, and ones for which we currently lack most of the important data. Most importantly, we need to know how the animal itself weights the different experiences – which negative experiences are worst, and to what degree different positive experiences may balance them out. There are a number of methods that we may use to assess the welfare of wild animals (e.g. Harvey et al. 2020), and in a future paper we will take a closer look at some specific methods that may be suitable for these ends. Which methods will work best will depend on the context of measurement, and the types of answers we are looking for. However, what is important is to establish how the different experiences of an animal’s life interact and trade off to form a total welfare experience, and whether this will be overall positive or negative. Importantly, more data is urgently needed to settle the matter and allow us to move forwards with planning effective strategies for assistance where required. We should not assume too much before we know more. It is thus important that animal welfare science expand its scope to include all sentient animals - whether in captivity or in the wild - and of particular importance that the science of wild animal welfare encompasses investigation of positive welfare states alongside possible sources of suffering. Only then will we be able to accurately judge whether, when, and how we might intervene to improve wild animal lives.
